# A Strategy for Eliciting Antibodies against Cryptic, Conserved, Conformationally Dependent Epitopes of HIV Envelope Glycoprotein

**DOI:** 10.1371/journal.pone.0008555

**Published:** 2010-01-05

**Authors:** Hanna C. Kelker, Vincenza R. Itri, Fred T. Valentine

**Affiliations:** 1 Department of Medicine, New York University School of Medicine, New York, New York, United States of America; 2 Department of Microbiology, New York University School of Medicine, New York, New York, United States of America; University of California San Francisco, United States of America

## Abstract

**Background:**

Novel strategies are needed for the elicitation of broadly neutralizing antibodies to the HIV envelope glycoprotein, gp120. Experimental evidence suggests that combinations of antibodies that are broadly neutralizing *in vitro* may protect against challenge with HIV in nonhuman primates, and a small number of these antibodies have been selected by repertoire sampling of B cells and by the fractionation of antiserum from some patients with prolonged disease. Yet no additional strategies for identifying conserved epitopes, eliciting antibodies to these epitopes, and determining whether these epitopes are accessible to antibodies have been successful to date. The defining of additional conserved, accessible epitopes against which one can elicit antibodies will increase the probability that some may be the targets of broadly neutralizing antibodies.

**Methodology/Principal Findings:**

We postulate that additional cryptic epitopes of gp120 are present, against which neutralizing antibodies might be elicited even though these antibodies are not elicited by gp120, and that many of these epitopes may be accessible to antibodies should they be formed. We demonstrate a strategy for eliciting antibodies in mice against selected cryptic, conformationally dependent conserved epitopes of gp120 by immunizing with multiple identical copies of covalently linked peptides (MCPs). This has been achieved with MCPs representing 3 different domains of gp120. We show that some cryptic epitopes on gp120 are accessible to the elicited antibodies, and some epitopes in the CD4 binding region are not accessible. The antibodies bind to gp120 with relatively high affinity, and bind to oligomeric gp120 on the surface of infected cells.

**Conclusions/Significance:**

Immunization with MCPs comprised of selected peptides of HIV gp120 is able to elicit antibodies against conserved, conformationally dependent epitopes of gp120 that are not immunogenic when presented as gp120. Some of these cryptic epitopes are accessible to the elicited antibodies.

## Introduction

The search for a vaccine to prevent the acquisition of HIV infection is complicated in part by the lack of a convincing immunological correlate of protection against HIV. Ideally one would like to elicit both HIV-specific broadly neutralizing antibodies (Abs) and T cell-mediated immunity, although optimal immunization strategies for these two types of immune responses may differ. To date, immunization strategies inducing cytotoxic CD8 T cells (CTLs) have impressively mitigated the severity of infection in nonhuman primates following challenge with SIV or SIV with an HIV envelope (SHIV), but have not prevented the acquisition of infection.

During the course of natural HIV infection sequential Abs develop that transiently neutralize existing viral variants, only to select resistant escape variants, indicating that Abs can inhibit HIV replication *in vivo* [1], [2], [3], [4]. However most Abs induced early in the course of infection are directed primarily against epitopes on the envelope glycoprotein, gp120, that are not conserved, and are expressed only on limited isolates within the quasispecies infecting the patient, allowing for selection of escape mutants [1], [2], [3], [4], [5], [6]. A small number of monoclonal antibodies (MAbs) that are able to broadly neutralize multiple subtype B isolates and some other group M isolates have been isolated by sampling the B cell repertoire of HIV infected patients [7], [8], [9], [10], [11], [12], [13], [14], [15], [16], [17]. The identification of these MAbs, directed against epitopes in the CD4-binding region, a mannose epitope on the glycosylated surface of gp120, and the membrane-proximal region of gp41 [18] demonstrates that some patients have B cell clones capable of making broadly neutralizing Abs. These MAbs have provided insights into the structure and function of gp120 [19], [20], [21], [22], [23].

As a proof of principal, the passive intravenous administration of a mixture of these human broadly neutralizing MAbs to Rhesus macaques prior to intravenous or intravaginal challenge with SHIV indicates that the presence of neutralizing Abs prior to exposure can prevent infection [24], [25], [26], [27], [28], [29], [30].

More recently antisera from a number of HIV-infected individuals, usually obtained late in the course of infection, have been described that are broadly neutralizing [31], [32], [33] and current studies are finding that the neutralizing Abs in these sera are directed against a variety of epitopes on gp120, including some in the CD4 binding region and in the co-receptor binding regions [31], [32], [33]. The recent cloning of single cell immunoglobulin cDNAs from 500 gp140 - binding memory B lymphocytes from HIV-infected patients with low viral loads, detected several neutralizing Abs from individual patients binding to multiple epitopes [16]. These observations are consistent with the existence of a variety of potential neutralizing epitopes on gp120. Evidence for the presence of multiple neutralizing epitopes recognized by Abs in infected individuals is reviewed elsewhere [5]. It should be appreciated that these human Abs have been induced by infection, and Abs elicited by immunization may be directed against different epitopes.

Although broadly neutralizing Abs can be formed in humans, and under specific experimental conditions are able to prevent infection in nonhuman primates, successful strategies for eliciting these or similar Abs by immunization have not yet been identified. Immunization of experimental animals or humans with recombinant, monomeric gp120 elicits antisera that are neither broadly neutralizing nor protective [34], [35], [36], although occasional neutralizing MAbs have been detected in these animal antisera [37].

Determinants of the CD4 and chemokine receptor binding sites, and those that permit the conformational changes required for viral entry should be conserved. The large conformational changes occurring upon CD4 binding [38] and the probable lack of a fixed conformation of unbound gp120 illustrate challenges presented by this target [39]. Immunization with stabilized trimeric gp120, with gp120 conformationally altered by sCD4 to expose buried epitopes, by decreased glycosylation, or with gp120 that has been engineered to display conserved epitopes often have been immunogenic, but with few exceptions all have failed to elicit Abs that are broadly neutralizing [40], [41], [42], [43], [44]. Molecular mimics of epitopes recognized by the existing neutralizing MAbs against gp120 have been selected by these MAbs, but most have failed to elicit similar Abs [45], [46], although screening of random peptide phage-displayed libraries, including conformational epitopes, with antiserum from a Rhesus monkey infected with a Clade C SHIV identified epitopes that induced neutralizing antibodies in mice primed with DNA encoding SHIV *env* [47]. Recently MAbs were isolated from a clade A infected individual that recognize broadly neutralizing conformational epitopes on gp120 only when in the trimeric form [17]. Conformationally dependent epitopes should generally be more highly conserved than those defined by a linear sequence of contiguous amino acid residues, and several conserved conformational epitopes have been documented, including some in the V3 loop, against which Abs are neutralizing [10], [48]. To be broadly neutralizing, an Ab should bind to a conserved epitope with a relatively high affinity. MAbs directed against an artificial FLAG epitope inserted into a part of the V4 loop of gp120, neutralized viruses, and the ease of neutralization by the anti-FLAG MAb was a function of the affinity of the MAb to mutants of the inserted FLAG [49]. Subsequent experiments indicate that the site of insertion of the tag may significantly influence the infectivity of the virus, and indicate the exact positioning of the tag may influence the ability of Abs to neutralize *via* this tag [50]. It has been suggested that conserved, conformationally dependent epitopes of gp120 are poorly immunogenic and/or inaccessible to Abs [51], [52]. We postulate that determinants exist on gp120 that are cryptic, in that they are not immunogenic when presented in the form of the whole glycoprotein, even though they are accessible to Abs should they be elicited. Some of these may be targets of known broadly neutralizing Abs, others may be targets of Abs not yet identified.

Strategies are needed for identifying additional cryptic, conserved epitopes, for eliciting antibodies to these epitopes, and determining whether these epitopes are accessible to antibodies. We have demonstrated that murine antisera recognizing cryptic, conserved conformationally dependent epitopes of HIV gp120 can be elicited by immunization with single sequence, multiple copy peptides (MCPs), also called MAPS, consisting of 8 identical peptide chains coupled into a branched structure *via* a core using the α and 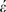 amino groups of lysine [53], [54]. These constructs have a molecular weight of up to 20,000 D, and enable one to immunize against a specific peptide without eliciting antibodies to large numbers of non-conserved sequences as occurs after immunizing with gp120, and without coupling it to a carrier protein, which might dominate the immunogenicity of the peptide, alter the immunogenicity of the peptide [55] or elicit lower Ab titers than achieved with the MCP [54]. We observed that the resulting whole antisera derived from the immunized animals bind to native but not fully denatured gp120, indicating that these immunogens focus an immune response on conformationally dependent epitopes in gp120 rather than contiguous linear epitopes [53]. The use of MCPs of a selected sequence can elicit antisera against conformationally dependent cryptic epitopes of gp120 formed by that sequence that cannot be elicited by immunization with the intact glycoprotein. Immunization with the homologous monomeric peptide does not elicit Abs against the epitopes on gp120 recognized by the anti-MCP sera even though high titers of Abs to the monomeric peptide are formed [53]. This represents a strategy for eliciting Abs to conserved conformationally dependent epitopes of gp120, against which Abs are not induced by immunization with gp120 itself.

We now present data on the functional properties of antisera and MAbs elicited in mice by three MCPs composed of two related amino acid sequences near the CD4-binding region and bridging sheet of gp120, encompassing residues 419–439, and 426–441, and a third conserved structure with a less conserved sequence, residues 363–384, that forms part of the CD4 binding pocket. The whole antisera as well as MAbs bind to native but not to completely denatured gp120, indicating that the majority of Abs in sera elicited by MCPs recognize secondary structures of the glycoprotein that are also expressed and accessible or partially accessible on native gp120. The Abs bind to gp120 on the surface of infected CD4 cells, and recognize cryptic epitopes not recognized by antisera elicited by gp120, or present at detectable levels in pooled human anti-HIV serum immunoglobulin (HIVIG). MAbs elicited by these MCPs bind to gp120 at half saturation concentrations of 2 nM or less, and one MAb has moderate neutralizing activity against an easily neutralizable clade B isolate SF162 [56], [57]. This strategy enables one to identify new potential epitopes in selected domains of gp120, elicit Abs against these epitopes, and use these Abs to assess whether the epitopes are accessible in monomeric and oligomeric gp120.

## Results

### Immunogenicity of MCPs

Because of their larger molecular weight and constrained secondary structure, immunization of mice with MCPs representing epitopes of gp120, elicits high titered antisera recognizing native recombinant gp120, whereas immunization with the homologous monomeric peptides does not, even though in some strains of mice high titers of Abs to the monomer peptide are formed (Table 1 and ref. [53]. Antibodies elicited by a monomer do cross-react at lower titers with MCPs of the same sequence (not shown), but MCPs display additional secondary structures or epitopes not present on the homologous monomer [53]. The 419–439 and 426–441 sequences are in the β20–β21 hairpin domain, which changes conformation within the gp120 molecule upon CD4 binding, and is part of the bridging sheet involved in interacting with the chemokine receptor during HIV entry [19]. Antisera elicited by 363–384 MCP, a sequence contributing to the CD4 binding pocket, bind to native gp120 at a lower titer than antisera induced by the other MCPs. The 419–439 MCP elicited antibodies against gp120 in C57Bl/10, BALB/c, C3H/HeJ, and SWR strains of mice. The 426–441 MCP elicited antibodies against gp120 in C57Bl/10 and BALB/c, but not in C3H/HeJ, and the 363–384 MCP only in C3H/HeJ mice. Although immune responses to the MCPs differ in the three inbred strains of mice, these 3 MCPs elicited Abs to gp120 in rabbits and in guinea pigs (not shown).

**Table 1:** MCPs and not the homologous monomeric peptides elicit antibodies that recognize envelope glycoprotein.

Antisera elicited by	Antibody titer to		
	gp120_MN_	Homologous MCP	Homologous monomer
419–439 MCP	36,000	850,000	4,800
419–439 monomer	440	610,000	250,000
426–441 MCP	120,000	1,800,000	3,200
426–441 monomer	95	50	<40
363–384 MCP	680	69,000	44,800
363–384 monomer	<20	<50	<50

BALB/c mice were immunized with 419–439 or 426–441 MCPs or with monomers, while C3H/HeJ were immunized with 363–384 MCP and monomer. Sera were collected after the second boost and antibody titers were determined.

### The Antisera Recognize an Epitope on gp120 Similar to the MCP Immunogen, and Antisera to the 419–439 or 426–441 MCPs Bind to a Shared Epitope

**Figure 1:**
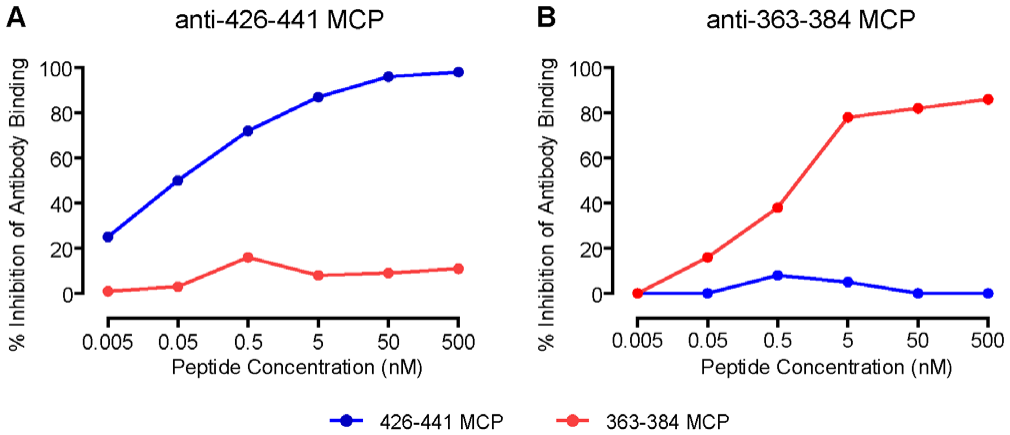
Binding of anti MCPs sera to gp120 is blocked by preincubation with the specific MCP immunogen. (A) Antiserum to 426–441 MCP, (B) Antiserum to 363–384 MCP. Sera derived from 426–441 MCP immunized C57Bl/10 mice (diluted 7250 fold) or from 363–384 MCP immunized C3H/HeJ mice (diluted 140 fold) were incubated with MCPs. Binding of antibodies to gp120 was determined by ELISA.

To ascertain that all Abs in the antisera elicited by MCPs are binding to epitopes on gp120 similar to the MCP, increasing concentrations of MCPs were incubated in solution with the Abs prior to incubating with gp120. In these competition ELISA experiments, MCPs inhibit the binding of Abs to gp120 in a concentration-dependent and antigen-specific manner. This specificity is demonstrated both for several polyclonal antisera elicited by MCPs (Figure 1A and B) and for MAbs (not shown).

**Figure 2:**
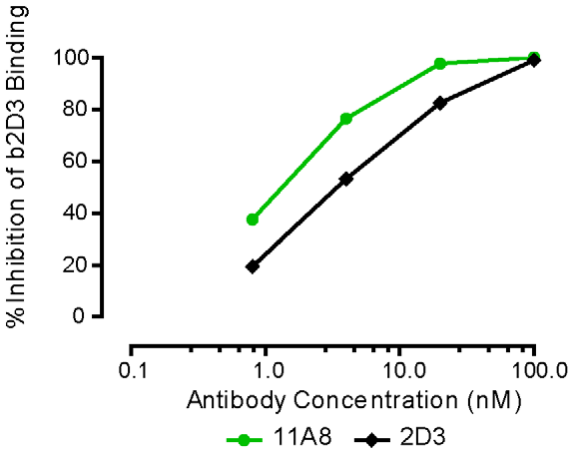
MAbs 11A8 and 2D3 compete for binding to gp120. Binding to gp120 of biotinylated 2D3 in the presence of unconjugated MAbs 2D3 or 11A8 was evaluated in ELISA. Concentration of biotinylated 2D3 in the assay was 1.2 nM, concentrations of competing, unconjugated MAbs 2D3 and 11A8 were as indicated.

Experiments in which the 419–439 MCP was synthesized in successively shorter lengths by eliminating amino-terminal residues, revealed that the dominant epitope recognized by the antisera and MAbs elicited by the 419–439 MCP was formed by residues in the carboxy end (data not shown). Therefore we investigated in parallel the immunogenicity of MCPs of the 419–439 and 426–441 sequences. The latter sequence adds 2 residues at the carboxy end and removes 7 residues from the amino end. The 426 MCP is more immunogenic, eliciting higher titers of antibodies to gp120, and to both 426 and 419 MCPs (not shown). As anticipated, antisera elicited by each of these MCPs bound to both MCPs and the respective MAbs (419 MCP MAb 2D3, and 426 MCP MAb 11A8) cross compete for binding to gp120 (Figure 2).

### Antibodies Elicited by MCPs Recognize Specific Conformationally Dependent Epitopes

**Figure 3:**
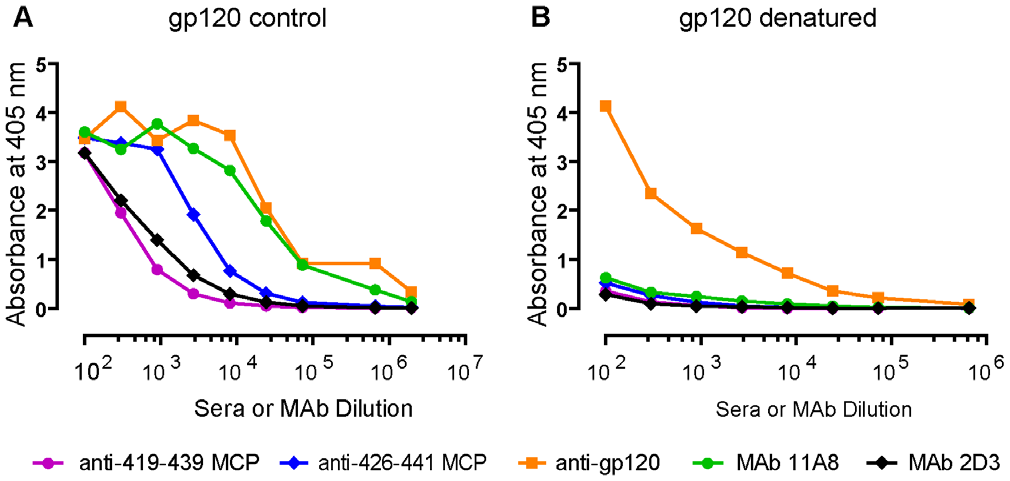
Antibodies elicited by MCPs fail to recognize denatured gp120. Recombinant gp120_MN_ or RCM gp120_MN_ were either maintained at 4° (control) or were heated for 5 minutes at 95° with 1 mM DTT, 0.1% SDS (denatured). Gp120 was then immobilized in ELISA wells using Ab D7324 and binding of antibodies was determined. Antigens captured on the plate were as follows: (A) gp120 (control); (B) gp120 (denatured). MAbs: 11A8 and 2D3, antisera to MCP 419 and to MCP 426 were all derived from C57Bl/10 immunized mice and antisera to gp120 was derived from C3H/HeJ mice. All sera and MAb preparations were diluted as indicated in the Figure. Protein concentration of the stock of undiluted MAb preparations used was, respectively: 1.96 mg/ml (MAb 11A8), and 0.5 mg/ml (MAb 2D3).

In order to assess the extent to which antisera elicited by MCPs bind to linear contiguous epitopes or conformationally dependent epitopes, antisera to 3 MCPs were examined for their binding to “native” and to completely denatured recombinant gp120. In the replicate experiments over a range of dilutions, antisera elicited by MCPs 419–439 and 426–441 bind to native but not denatured gp120 (Figure 3, Table 2), indicating that the majority of Abs in these antisera elicited by the MCPs bind to conformationally dependent epitopes, and also that these epitopes are accessible to the Abs. In contrast, antisera to gp120 also contain Abs binding to denatured gp120 (Figure 3B) in addition to Abs against conformationally dependent epitopes. Antibodies obtained from HIV infected individuals also shared these characteristics [58]. Although titers of 363–384 antisera against native gp120 were clearly lower than titers elicited by the other MCPs (Table 1), the binding of 363 antisera to completely denatured gp120 also was decreased by 80 to 100% in multiple experiments, in parallel with the loss of binding observed with antisera elicited by other MCPs (Table 2). A 419 MCP MAb (2D3) and 426 MCP MAb (11A8) also bind to native but not denatured gp120 (Figure 3).

**Table 2:** Recognition of native gp120_MN_ and of RCM** gp120_MN_ by antisera elicited by MCPs.

Antisera to	Antibody titer to gp120			Titer ratio
	Native	Denatured*	RCM**	(RCM/Native)
363–384 MCP	210	<50	25,000	119.0
419–439 MCP	100,000	<50	140,000	1.4
426–441 MCP	300,000	2,500	400,000	1.3

* gp120 was denatured as described in the legend of Figure 2.

** RCM, reduced, carboxymethylated gp120_MN_.

Sera to 363–384 MCP were harvested after the third boost, and sera to 419–439 MCP or 426–441 MCP after the second boost. Antisera to MCPs 363 and 419 were obtained from C3H/HeJ mice and to 426 MCP from C57Bl/10 mice. ELISA wells were coated directly with gp120_MN_ native, gp120 denatured (as described in the legend of Figure 3) or with RCM gp120_MN_ and antibody titers were determined.

However, if antisera elicited by MCP 363 are assessed for their binding to reduced carboxy-methylated (RCM) gp120 in which S-S bonds are disrupted [59] the binding of anti-363–384 sera is enhanced 100 fold as compared with native gp120 (Table 2). In contrast, the binding of the other anti-MCP sera to RCM gp120 is not significantly affected (Table 2). Since the 363 MCP antisera do not bind to completely denatured gp120, the enhanced binding to RCM gp120 suggests that the majority of antibodies in the 363 antisera are directed against a residual secondary conformational epitope on gp120 that is relatively inaccessible in the native molecule, explaining the lower titer to native gp120. The epitopes recognized by the 419 MCP and 426 MCP antisera as well as MAbs (not shown) are accessible on both native gp120 and RCM gp120 (Figure 3, Table 2). This data also indicates that some secondary structure remains in RCM gp120, and that some of these remaining structural epitopes are also accessible on native gp120. We conclude that the 419–439/426–441 epitopes in native gp120 are accessible to Abs, but that the majority of epitopes in native gp120 recognized by Abs elicited by the 363–384 MCP are not accessible to Abs.

### MCPs Elicit Antibodies to Cryptic Epitopes

**Figure 4:**
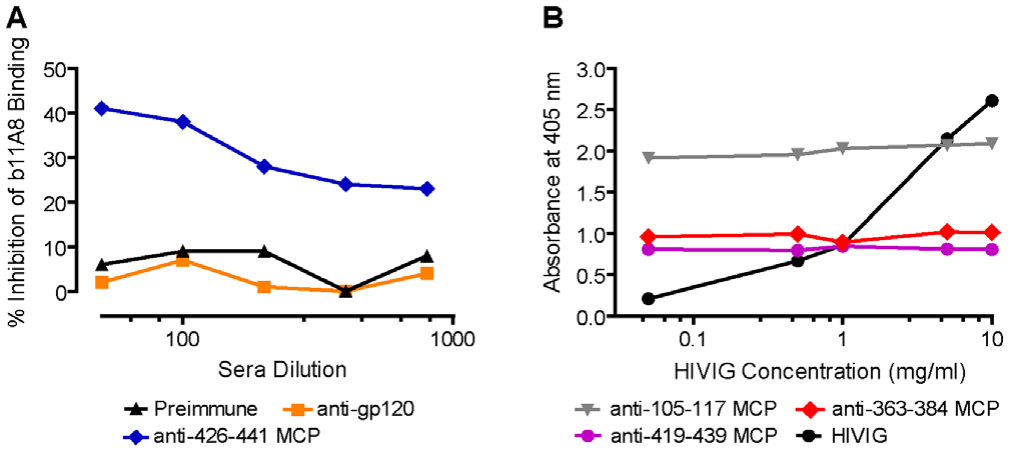
MCPs elicit antibodies to cryptic epitopes of gp120. (A) Binding of MAb 11A8 to gp120 is not inhibited by antisera to gp120. Competition for gp120_MN_ binding of biotinylated MAb 11A8 and murine sera was evaluated in ELISA as described in Materials and Methods. All competing sera were obtained from C57Bl/10 mice prior to immunization (preimmune), from mice immunized with 426–441 MCP (130, 000) or with rgp120MN (550, 000). Titers of sera antibodies recognizing gp120 are listed in parenthesis. Results are expressed as % inhibition of biotinylated 11A8 binding to gp120 by competing antibodies. (B) HIVIG does not inhibit the binding to gp120 of murine antibodies elicited by MCPs 419–439, 363–384 or 105–117. Competition between HIVIG and anti-MCP sera for binding to gp120 was assayed by ELISA as described in Materials and Methods. Gp120 was incubated with HIVIG at the indicated concentrations and the binding of anti-MCP sera (colored lines) from C3H/HeJ mice (anti-MCPs 105–117 and 363–384), C57Bl/10 mice (419–439 MCP) or of HIVIG (shown as black line) was determined. Results are expressed as absorbance at 405 nm.

MCPs can elicit antibodies against cryptic epitopes that are not immunogenic when presented in the gp120 protein, and against which Abs may not develop during HIV infection in humans. Antisera elicited by immunization with rgp120 and antibodies present in hyper-immune human IgG (HIVIG) bind weakly to MCPs 419–439, 426–441 or 363–384 (Table 3). Furthermore, preincubation of gp120 with murine anti-gp120 sera, elicited in the same strain of mice, up to saturating concentrations does not block the binding of biotinylated 426 MCP MAb (11A8), whereas the binding of this MAb is inhibited by antisera elicited by MCPs derived from this epitope (Figure 4A). Taken together, these two types of experiments indicate that the administration of an epitope in the form of a MCP can elicit antibodies accessing cryptic epitopes on gp120 that are not immunogenic when presented in the intact glycoprotein. In a parallel type of experiment, preincubation of gp120 with increasing concentrations (even beyond data shown) of human HIV Immunoglobulin (HIVIG) does not inhibit the binding to gp120 of antibodies elicited by 3 different MCPs (Figure 4B) even though the HIVIG binds strongly, suggesting, but not proving that antibodies against these epitopes are not present at detectable levels in humans infected with HIV.

**Table 3:** Antibodies to MCPs are not elicited by immunization with gp120 or by HIV infection.

Antibody Source	Antibody titer to			
	gp120_MN_	419–439	426–441	363–384
Murine antisera to gp120_MN_ *	1,970,000	160	200	100
HIV Immunoglobulin**	1,250,000	1,600	1,300	900

ELISA wells were coated with gp120_MN_ or with MCPs listed in the Table, and antibody titers were determined.

* Sera were obtained from C3H/HeJ mice immunized with gp120_MN_.

** HIV Immunoglobulin, purified from pooled HIV+ human plasma.

### MCPs Elicit a Relatively Focused Range of Antibody Specificities

**Figure 5:**
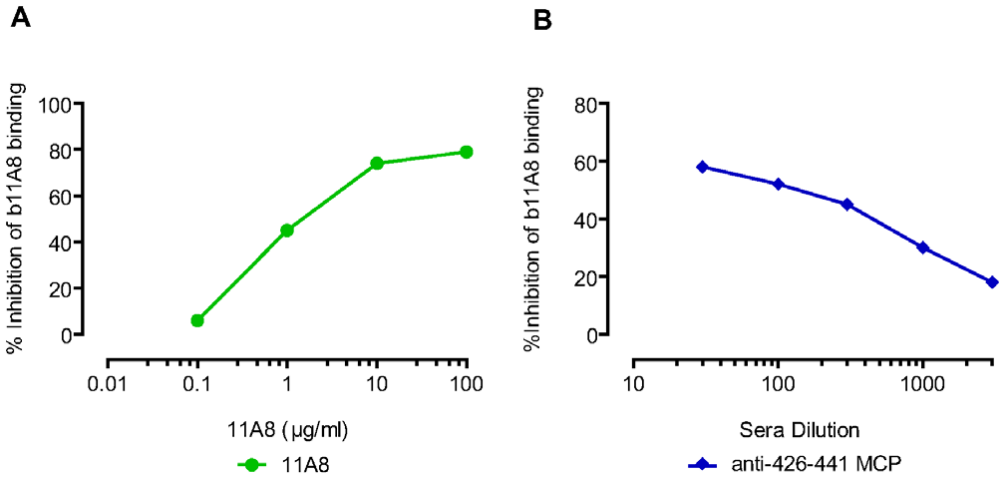
Determination of antibodies in 426–441 MCP immune sera that can compete with 11A8 for gp120 binding. Competition of antibodies and biotinylated 11A8 (0.02 µg/ml) for binding to gp120_MN_ was determined in ELISA as described in Materials and Methods. Source of competing antibodies: (A) Purified, unconjugated 11A8 - used as a standard; (B) Sera harvested from mice immunized with 426–441 MCP. MAb 11A8 and 426–441 MCP sera were derived from C57Bl/10 mice. Results are expressed as percent inhibition of biotinylated 11A8 binding by competing antibodies.

Peptide immunogens composed of multiple copies of the same sequence should elicit a relatively restricted range of Abs focused on the related epitopes presented by the MCP. Data presented in this paper emphasize that the majority of Abs present in the whole antisera elicited by a MCP recognize conserved, cryptic, conformationally-dependent epitopes, yet experiments on the functions of these antibodies often employ a MAb in order to avoid certain nonspecific effects of mouse serum. In order to estimate the approximate concentration of antibodies in an antiserum that have the same specificity as a given MAb isolated from mice immunized with the MCP, we measured the dilution of antiserum required to compete with the binding to gp120 of a biotinylated MAb, as compared with the MAb itself. The binding of the biotinylated MAb is blocked by the homologous unconjugated 11A8 MAb (Figure 5A). The amount of homologous unlabeled MAb required to block the binding of biotinylated MAb by 50% was determined from the linear portion of a standard curve. Data in Figure 5B in which antisera elicited by MCP 426 are examined for their ability to inhibit the binding of biotinylated MAb 11A8 to gp120, indicate that a 1∶300 dilution of antiserum gives the same inhibition of binding to gp120 as 0.6 to 1 µg/ml of the MAb. These data combined with that obtained from additional 426 MCP sera (not shown) suggest that the antisera contain antibodies, focused on the same epitope, equivalent to the MAb 11A8 at concentrations of 240 to 400 µg/ml, although the relative affinities of the antibodies in the sera are undetermined.

### Monoclonal Antibodies (MAbs) Elicited by MCPs Bind to gp120s from Multiple Clades with Varying Affinities

The MCPs were constructed from a subtype B sequence, and in repeat experiments MAbs elicited by the MCPs 419 or 426 bind to clade B gp120 with affinities ranging from 1 to 100 nM, as estimated by the concentration required to achieve half saturation of binding. The sequence 419–441 of gp120 is relatively conserved, but may vary by a few residues within clade B isolates. Although the overall tertiary structures of gp120s are assumed to be essentially identical since gp120 of multiple genotypes, and the envelope protein of SIV, must bind to CD4 and chemokine receptors, and share domains and many conserved sequences [19], [39], [51], [60]. These MAbs bind to gp120s from other clades with lower affinities ranging from 80 to 480 nM undoubtedly reflecting differences in the primary sequences (Table 4). MAb 11A8 binds to gp120 of clade B significantly less than MAb b12 (Table 5) but in the same range.

**Table 4:** MAbs elicited by 426–441 MCP (11A8) or 419–439 MCP (2D3) bind to recombinant gp120s derived from HIV-1 clades B, C, E, and A/E.

MAb	K_D_ (nM) for gp120								
	Cl. B				Cl. C			Cl. E	Cl. A/E
	MN	SF162	BAL	SF2	CN54	96ZM651	CM	93TH975	244
11A8	0.56	0.15	100	1.7	220	140	210	240	300
2D3	9	1.4	43	12.5	155	80	480	160	370

* K_D_ the concentration of MAbs resulting in a half saturation of gp120 binding. Recombinant gp120s derived from different HIV isolates (from clades B, C, E and A/E, as listed in the Table) were captured in ELISA wells coated with antibody D7324 and MAb binding was determined by ELISA.

**Table 5:** MAb 11A8 detects HIV on the surface of cells infected by HIV isolates from clades A, B, D, and E.

HIV-1 isolate	Clade	Antibody binding (% positive)				
		IgG_1_κ*	11A8	IgG_1_b12	F 105	HIVIG
92UG029	A	0.5±0.3	13±2	33±19	1±1.4	22±10
301596	B	0.6±0.3	24±9	63±8	22±11	39±10
UG/93/086	D	0±0	19±0	6±7	8±11	29±12
CMU 08	E	1±0	14±3	n.d.	n.d.	15±9

* Murine IgG_1_ *κ* was used as an isotypic control for MAb 11A8.

CEM-4 cells were infected with HIV isolates from different clades. Cells were incubated with the antibodies at saturating concentrations and the antibody binding was determined by flow cytometry using the appropriate secondary antibodies. Results are expressed as % of cells fluorescing.

### 11A8 MAb Elicited by MCP Binds to HIV Infected Cells

**Figure 6:**
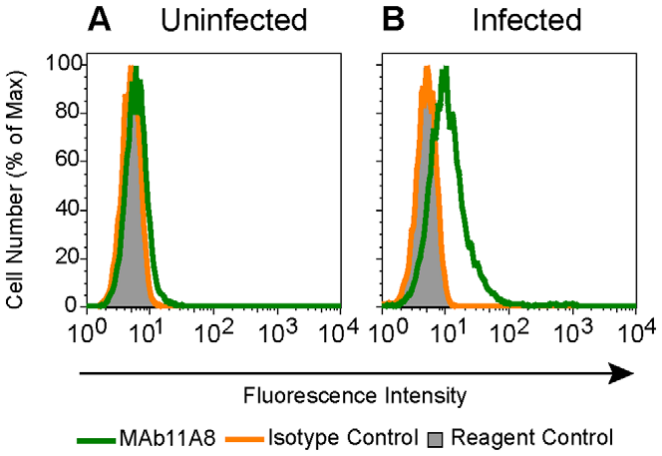
A MAb elicited by 426 MCP (11A8) recognizes HIV-1 on the surface of infected cells. (A) Uninfected CEM-4 cells, (B) CEM-4 cells infected with HIV-1 clade B. Cells were incubated, as indicated, with 5 µg of 11A8 or of anti-TNP (isotypic control), or with no primary antibody (reagent control). Antibody binding was detected using PE-conjugated secondary antibody and measured by flow cytometry. Flow histogram plots are displayed.

**Figure 7 pone-0008555-g007:**
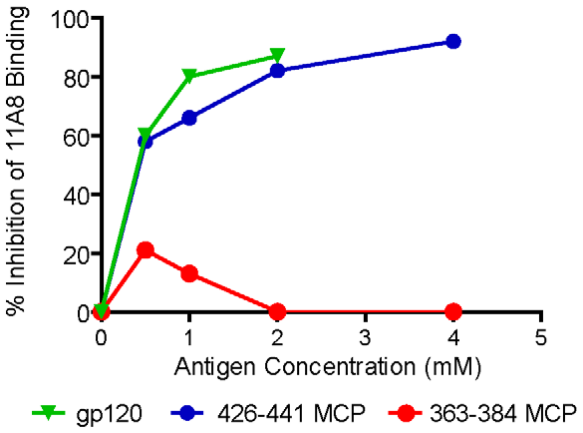
11A8 binding to HIV on infected cells is blocked by gp120 and by homologous MCP. 11A8 was incubated for 30 min at 37° with 426–441 MCP, 363–384 MCP or with gp120 in 2% FCS RPMI and subsequently the binding of 11A8 to HIV-1 clade B infected CEM-4 cells was assayed. Final concentration of 11A8 in the binding assay was 0.5 µM, and concentrations of competing antigens were as indicated. MAb 11A8 binding to cells was determined by flow cytometry and % inhibition of MAb binding by antigens was calculated.

**Figure 8:**
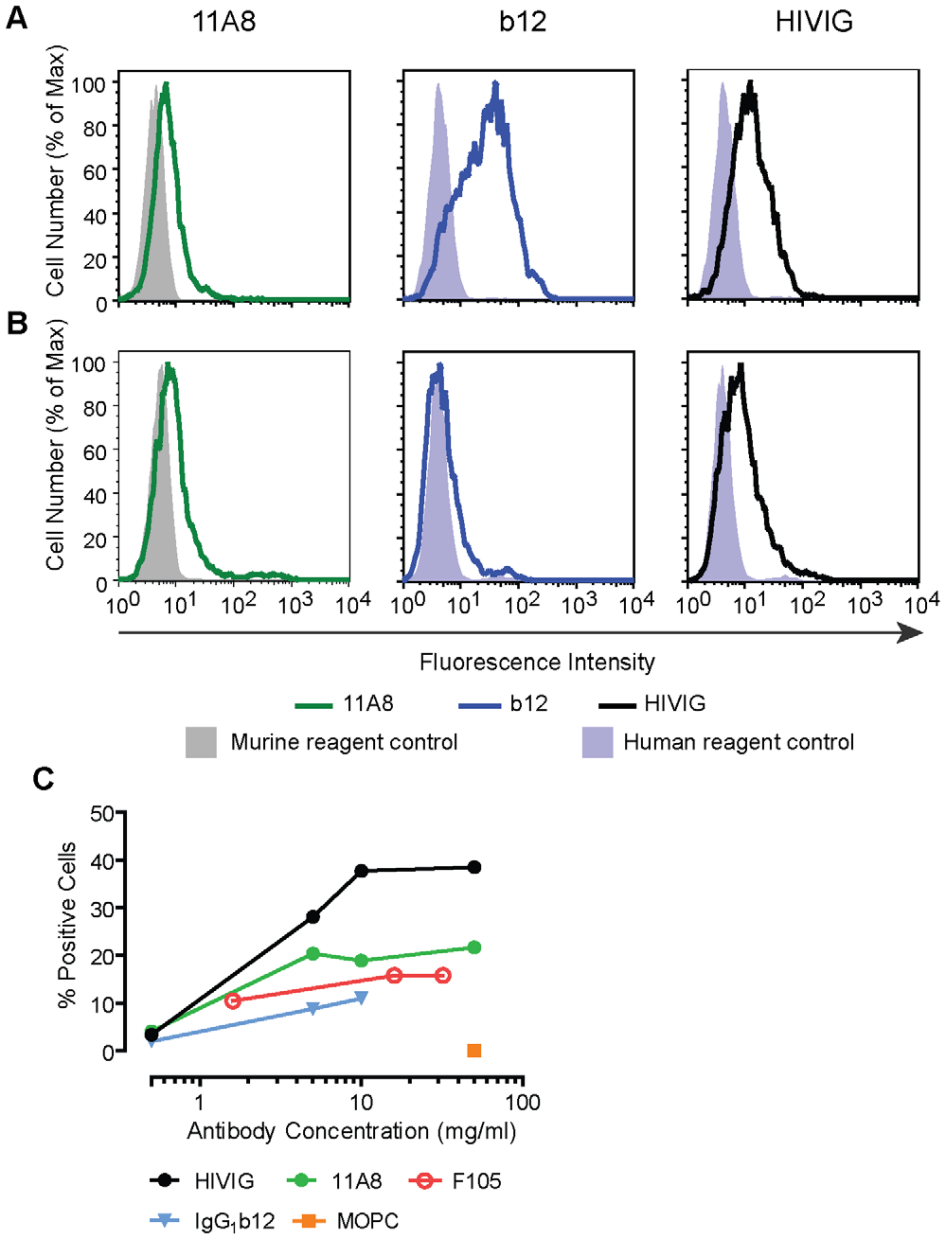
11A8 binds to cells infected by a clade D HIV isolate. CEM-4 cells infected with (A) HIV-1 clade B or (B) with clade D isolate, were incubated with MAbs 11A8, b12 or with HIVIG at 10 µg/ml. Antibody binding to cells was detected using the appropriate secondary antibodies. The flow histogram plots of the fluorescence intensity of antibody binding to cells are displayed. (C) Antibody binding to clade D infected cells: concentration dependence. Clade D infected cells were incubated with MAbs 11A8, MOPC (murine isotypic control), b12, F105 or with HIVIG at the indicated concentrations. Percentage of cells binding antibody to the cell surface was determined by flow cytometry.

Gp120 exists as a trimer on the viral membrane and on the surface of infected cells [23], [60], and this trimeric structure might render some epitopes present on monomeric gp120 inaccessible to antibodies. The recognition of gp120 in an oligomeric form by anti-426 MCP MAb (11A8) was examined by measuring its binding to HIV-infected cells using flowcytometry. The MAb 11A8 bound to a larger portion of cells infected with HIV-1 clade B, with higher mean channel fluorescence, than to uninfected cells (Figure 6). The specific binding of this antibody was inhibited in a dose-dependent manner by preincubation with gp120 or with the homologous but not heterologous MCP (Figure 7). The MAb also bound to cells infected with HIV from additional clades (Table 5), and bound with a greater intensity (mean channel fluorescence) and to more cells infected with an HIV-1 isolate from clade D than the positive control MAb b12, which is known to bind to oligomeric gp120 (Figure 8, Table 5). HIVIG bound to cells infected by HIV from each of these clades to approximately the same degree except for an isolate from clade E, suggesting that different gp120s are expressed to approximately the same degree on these infected cells. The murine isotypic control did not bind to infected cells. The binding of 11A8 and of b12 to infected cells does not represent binding of these MAbs to passively bound gp120 on cells since both MAbs 11A8 and b12 do not detect soluble rgp120 bound to uninfected CD4 positive cells (Table 6). In contrast, gp120 passively bound to uninfected cells is detected by human polyclonal antibodies in HIVIG (Table 6). The failure of the anti-426 MCP MAb to bind to gp120 complexed with CD4 on uninfected cells also indicates that the epitopes recognized by this antibody and by MAb b12 are not accessible when gp120 is bound to CD4.

**Table 6:** MAb 11A8 does not recognize gp120 passively bound to uninfected CEM-4 cells.

Antibody	Ab binding (% positive)	
	gp120_MN_	Control
None*	0.1	0.1
11A8*	1.2	0.9
None**	1.5	1.3
IgG_1_b12**	1.3	1.4
HIVIG**	96.2	1.3

CEM-4 cells were incubated for 2 h at 4°C with gp120_MN_ (0.24 µg/ml) or with no gp120 (control), washed, then incubated with the primary antibodies (10 µg/ml) listed in the Table. Antibody binding to cells was determined by flow cytometry. Results are expressed as % of cells fluorescing. Secondary antibodies:

* anti-mouse.

** anti-human.

### Interactions between Soluble CD4 and Anti-MCP Abs

**Figure 9:**
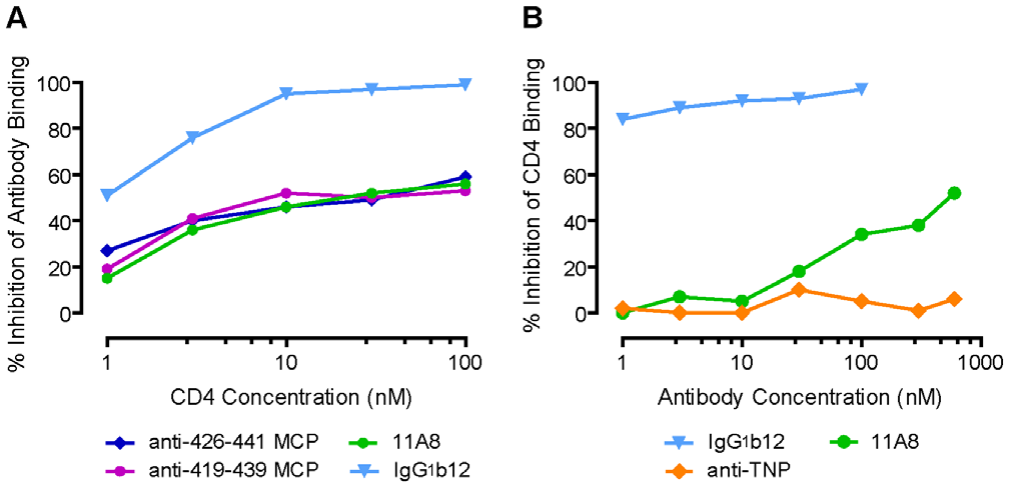
Antibodies elicited by MCPs 419 and 426 partially compete for CD4 binding to gp120. (A) CD4 inhibition of antibodies binding to gp120. (B) MAb 11A8 weakly inhibits CD4 binding to gp120. Reciprocal competition of CD4 and antibodies for binding to gp120 was evaluated by ELISA as described in Materials and Methods with gp120 captured on the antibody to the C-terminal sequence of gp120.

The sequences 419–439 and 426–441 are in the β20–β21 hairpin of the bridging sheet [19] and antibodies to these epitopes might influence the binding of sCD4, or alternatively, sCD4 might inhibit the binding of the antibodies, either by steric hindrance or by changing the conformation of the β turn. To examine these possibilities, gp120 was first incubated with sCD4, which at 10 nM blocked the binding of anti-MCP sera or MAb 11A8 by 50%. The same concentration of sCD4 blocked the binding of MAb b12 by 95% (Figure 9A). Reciprocally, MAb 11A8 first incubated with gp120, inhibited the subsequent binding of sCD4 by 34 to 50% only at high concentrations of 100 to 600 nM, while MAb b12 inhibited 80% at 1 nM (Figure 9B). As would be expected, a murine isotypic control MAb does not affect CD4 binding.

### A MAb Elicited by 426–441 MCPs Has Modest Neutralizing Activity against an Easily Neutralizable HIV Isolate from Subtype B

**Figure 10:**
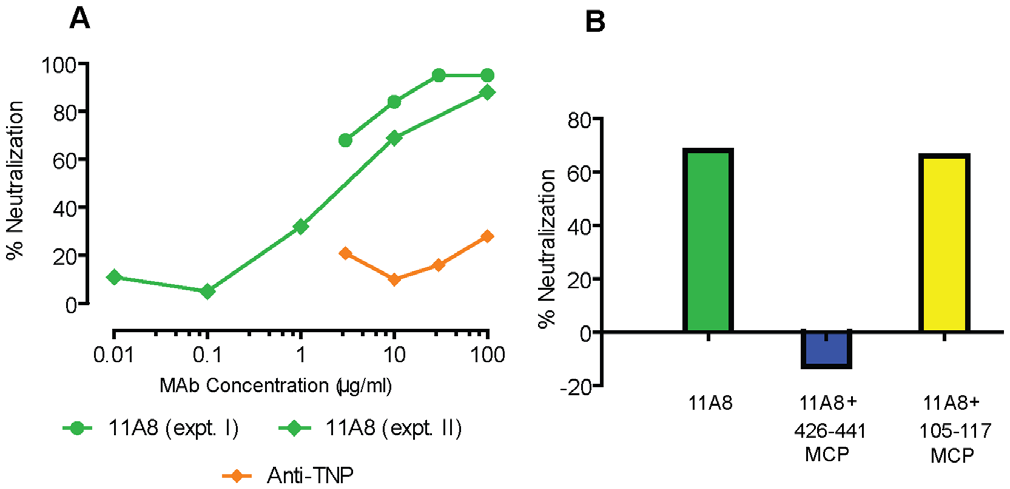
MAb 11A8 neutralizes SF162 pseudovirus. (A) Concentration curve of 11A8 and the murine isotypic control anti-TNP for SF162 pseudovirus neutralization. (B) Neutralization of SF162 by 11A8 is abolished by preincubation with the homologous MCP. MAb 11A8 was incubated before neutralization assay for 1 h at 37° with 426–441 MCP or 105–117 MCP or with medium (control). The final concentration of 11A8 in neutralization assay was 0.1 µM and of the MCPs was 1 µM.

To investigate an additional functional property of these MAbs, we examined the ability of the MAb with the highest affinity for gp120 (11A8) to neutralize the SF162 isolate, a strain know to be easily neutralized [56], [57]. MAb 11A8, elicited by immunization with 426–441 MCP, was able to neutralize SF162 by 30 to 65% at a concentration of 1 µg/ml and 80 to 90% at 30 µg/ml, using a luciferase construct expressing SF162 gp120 (Figure 10A). The neutralizing activity was blocked by preincubation of the MAb with the specific but not an unrelated MCP (Figure 10B). Other MAbs of lower affinity elicited by 419–439 MCP had little or no neutralizing activity (not shown).

## Discussion

The design of immunogens eliciting broadly neutralizing antibodies against HIV should be facilitated by approaches that focus the antibody response on selected, additional epitopes formed by conserved sequences or included in conserved domains, and recent studies suggest the existence of a number of additional neutralizing epitopes [5], [16], [31], [32], [33]. Strategies are needed for identifying additional cryptic, conserved epitopes, to devise immunogens eliciting Abs against these epitopes, and for determining whether these epitopes are accessible to antibodies. These epitopes are more likely to be conserved if they are conformationally dependent, and must be accessible to antibodies on oligomeric wild type gp120 [61]. In an attempt to develop such a strategy, we have explored the immunogenicity of Multiple Copy Peptides (MCPs) [53], [54]. We have reported previously on the immunogenicity of MCPs encompassing residues 105–117 and 419–439 of gp120, although in most previous experiments binding to gp160 rather than gp120 usually was measured [53]. We now describe the specificities and functional properties of the antisera and MAbs elicited by three different MCPs comprised of peptides from HIV gp120. In multiple immunizations we have elicited antisera with these properties to 4 out of 4 selected sequences, using MCPs representing 4 sequences in 3 different determinants on gp120 (reference 53 and Results section).

419–439 MCP and 426–441 MCP contain sequences from the β20–β21 hairpin of the bridging sheet region [19]. This determinant is in the same structure as that identified as binding neutralizing Abs recently detected in a broadly neutralizing human serum [33]. The sequence comprising the 363–384 MCP contributes a significant portion of the CD4-binding pocket, and contains conserved residues essential for CD4 binding [19], [62], [63]. The 363–371 and 425–430 sequences account for 57% of the total contribution of gp120 to the binding of CD4 [19]. The bridging region and CD4 binding pocket are logical targets for the development of neutralizing antibodies, and many naturally occurring antibodies are directed against the CD4 binding surface only some of which are neutralizing [12], [31], [32], [33].

The β20–β21 hairpin containing sequences 419–439 and 426–441 of the bridging sheet might be expected to undergo a conformational change upon CD4 binding, and preincubation of gp120 with sCD4 resulted in a modest inhibition of the binding to gp120 of antibodies to this epitope (Figure 9A). Reciprocally, preincubation of gp120 with high concentrations of MAb (11A8) to the 426–441 epitope resulted in a small inhibition of CD4 binding to gp120, far less than achieved by the MAb b12 (Figure 9B).

The binding to gp120 of antisera elicited by MCPs is blocked by the specific MCP used as the immunogen (Figure 1), indicating that the antibodies are specific for epitopes on gp120 that cross react or are conformationally compatible with those of the MCP. Data presented in additional experiments evaluating the ability of anti-MCP antibodies to bind to gp120 on infected cells or to neutralize HIV-1_SF162_ indicate that these functions also are inhibited by prior incubation of the antibodies with the MCP used as the immunogen.

MCPs elicit Abs to cryptic epitopes that are poorly immunogenic when native gp120 is used as an immunogen. This is based on observations that anti-gp120 sera and HIVIG bind poorly to MCPs 419–439, 426–441 or 363–384 (Table 3). The failure of antigp120 sera to block the binding to gp120 of a 426 MCP MAb (Figure 4A) suggests that the 11A8 epitope is cryptic in gp120, although the concentrations of Abs that might resemble the 11A8 MAb in antisera to gp120 is not known,

Similarly the failure of HIVIG to block the binding to gp120 of antisera elicited by MCPs (Figure 4B) suggesting that HIV-infected humans may not make detectable levels of antibodies against these epitopes. However the interpretation of this last experiment is difficult because the mixture of Abs in HIVIG also does not block the binding of murine anti-gp120 (not shown), suggesting that: a) at the concentration of the broadly reactive HIVIG employed the amount of potential competing Abs in HIVIG is too low to block binding, or b) the majority of epitopes recognized by murine and human B cells are sufficiently different that similar Abs cannot be detected in competition assays. It should be noted that the mixture of antibodies in HIVIG does provide this reagent with potent neutralizing activity.

These data suggest that the strategy of immunizing with MCPs may enable one to elicit antibodies to additional cryptic epitopes on gp120. The data also indicate that cryptic epitopes may be accessible to Abs elicited by MCPs even though they are not immunogenic when the intact protein is the immunogen.

Our data suggests that the antibodies in antisera elicited by MCPs are directed to conformationally dependent epitopes and not to linear contiguous epitopes (Figure 3A and B, Table 2). The observation that 363 MCP elicited lower titers of antibodies binding to native gp120 than 419 MCP or 426 MCP (Tables 1 and 2) was explained by subsequent experiments demonstrating that most of the antibodies induced by 363 MCP were directed against epitopes accessible in RCM gp120 in which S-S bonds are disrupted (Table 2).

By contrast, binding of antisera or MAbs elicited by MCPs 419 or 426 to RCM gp120 and native gp120 was similar, indicating that binding sites for the 418 or 426 epitopes are present in an accessible form in both native and RCM gp120. However, all of these antibodies failed to bind to fully denatured gp120, suggesting that residual secondary structures able to bind these antibodies are present in RCM gp120. It has been demonstrated previously that interactions between β sheets can drive partial folding of gp120 even after a critical S-S bond has been eliminated [64], [65]. This result suggests that the majority of antibodies elicited by the 363 MCP are directed against secondary structures that are inaccessible in the native gp120, but that some of the antibodies bind to more accessible epitopes in the CD4 binding region of native gp120, since the binding of these Abs is blocked by the 363 MCP immunogen and is lost when gp120 is completely denatured. The ability of 363 MCP to elicit some antibodies binding to more accessible epitopes in the CD4 binding region of native gp120 is of interest in light of the recent finding that some neutralizing antibodies in the sera of patients with broadly neutralizing activity are directed against epitopes in this region [32], [33], and that a broadly neutralizing Ab like b12 may initially bind to the epitopes around the binding pocket at low affinity and then bind the interior of the CD4 binding pocket [66].

Epitopes binding antibodies elicited by the 426 MCP also are accessible on oligomeric gp120 as indicated by the ability of the antibodies to bind to gp120 of budding virus on the surface of infected cells (Figures 6–8 and Table 5). Binding of an Ab to infected cells is frequently used as a surrogate for binding to gp120 in its trimeric form, although monomers and dimers may also be present on the surface of HIV-infected cells [61]. MAb b12 is known to bind to gp120 in its trimeric form, and MAb elicited by the 426 MCP also bound to cells infected with several subtypes of HIV in approximately the same range as b12, and bound at greater intensity to more cells infected with clade D virus (Figure 8). Control experiments demonstrate that the binding of 11A8 to infected cells is specifically blocked by preincubation of antibody with homologous MCP (Figure 7) and that 11A8 does not detect gp120 passively bound to CD4 on cells (Table 6). Similarly, the neutralizing activity of a MAb elicited by 426 MCP against the easily neutralized SF162 is inhibited by preincubation of the antibody with the immunogen (Figure 10).

The MCPs used as immunogens were constructed using sequences from Clade B isolates, and while the elicited antisera bind most avidly to gp120 of clade B viruses, they also bind at a lower affinity to rgp120 from other clades (Table 4). A MAb elicited by 426 MCP demonstrates a similar breadth of binding to oligomeric gp120 on cells infected with viruses from different clades, and actually detects virus at a higher intensity and on more cells infected with a clade D isolate than does the MAb b12 (Table 5 and Figure 8).

Our hypothesis is that in MCPs the peptides, consisting of 15 to 21 residues, when coupled at the carboxyl end to the lysine core and constrained by 7 adjacent chains, assume a conformation compatible with that in the intact gp120. These secondary structural epitopes are immunogenic when presented in MCPs and also are present and often accessible in native gp120, whereas antisera elicited by monomers do not bind to gp120. Antisera elicited by MCPs recognize native gp120 but not denatured gp120, and modulate functions of the native protein. The strong recognition of conformational epitopes and poor recognition of contiguous linear epitopes (as in native but not denatured gp120) is a property of the whole antisera as well as MAbs, and does not represent a selection of one MAb that was elicited by immunization with the MCP. While we do not know the total amount of Ab against gp120 elicited by immunization with the MCPs, the competition ELISA studies (Figure 5) indicate that the antisera contain substantial amounts of Abs equivalent to 11A8, focused on the same epitope.

The difficulty in eliciting broadly neutralizing Abs to gp120 has been attributed to either the lack of immunogenicity of conserved epitopes, or the inaccessibility of these epitopes to antibodies if they are formed (as reviewed in [61]. The experiments described provide examples of each of these explanations. Both the MCPs 419 and 426 and the 363 MCP elicit antibodies to epitopes that are not immunogenic when presented to the immune system as native glycoprotein, and while epitopes in native gp120 are accessible to the Abs elicited by MCPs 419 and 426, most (but not all) of the antibodies elicited by the 363 MCP are directed against epitopes not accessible in the native glycoprotein.

Many additional conserved cryptic epitopes may be immunogenic if presented in the form of MCPs, and some of them may also be accessible on native gp120. Once Abs are elicited by a new MCP, these Abs can be used as reagents to determine whether the new epitope is accessible on monomeric and trimeric gp120. Other conserved structures may not be immunogenic because they are recognized by the immune system as being self.

The 3 MCPs reported in this paper do not elicit broadly neutralizing antibodies, even though modest neutralizing activity has been observed, and broadly reactive binding to cells infected with different subtypes has been demonstrated. Although the amino acid sequence encompassing residues 426–441 differs by 3 residues between 426 MCP and the SF162, the MAb elicited by this MCP neutralized SF162. However, these experiments do provide a strategy for eliciting antibodies to other conserved and accessible epitopes on 120 that are not immunogenic when presented in the whole glycoprotein. Although regions of gp120 associated with critical conformational changes or with binding to cells should be targets for neutralizing antibodies, additional conserved, accessible epitopes in non-functional regions also should be identified and studied. A combination of several MCPs representing epitopes broadly conserved across genotypes of HIV-1 might be effective in a prime-boost immunization schedule to focus the immune response on conserved and otherwise cryptic epitopes. Recent, increasingly precise identification of epitopes recognized by broadly neutralizing MAbs identified by repertoire screening or by broadly neutralizing antibodies in the sera of many infected individuals [5], [16], [32], [33] should provide opportunities to evaluate the strategy of immunizing with specific MCPs resembling these epitopes.

## Materials and Methods

### HIV-1 gp120 Derived Peptides

Peptides derived from conserved domains of gp120 were synthesized as monomers, or high molecular weight Multiple Copy Peptides (MCP). Amino acid sequences of the peptides and their numerical location in gp120 (in parenthesis) as in the Los Alamos National Laboratory HIV Sequences Compendium were as follows: 1) RIKQIINMWQEVGKAMYAPPI (amino acids 419–439),

2) MWQEVGKAMYAPPIEG (amino acids 426–441),

3) QSSGGDPEIVTHSFNCGGEFFY (amino acids 363–384),

4) HEDIISLWDQSLK (amino acids 105–117). These amino acid sequences are identical to sequences in HIV-1 HXB2. The MCPs, containing 8 identical peptides covalently attached to a polylysine core through the triglycine spacers were synthesized as described previously [53], [54]. Briefly, 8 copies of identical amino acid sequence were manually synthesized on the core using solid phase peptide synthesis F-moc chemistry. The major peak was then HPLC purified using a C18 reverse phase column and peptide sequence verified by amino acid analysis. Peptides were repeatedly synthesized at Anaspec (San Jose, CA) and the 363–384 MCP was also synthesized at Louisiana State University Health Sciences Center Core Laboratory (New Orleans, LA). 363–384 MCPs obtained from both sources elicited similar immune responses. The MCPs are referred to in the text as: 1) 419–439 MCP or 419 MCP; 2) 426–441 MCP or 426 MCP; 3) 363–384 MCP or 363 MCP and 4) 105–117 MCP or 105 MCP.

### Recombinant HIV-1 Envelope Glycoproteins

gp120_CM_, gp120_CN54_, gp120_93TH975_, gp120_BAL_, gp120_96ZM651_, gp120_SF162_ were obtained from the NIH AIDS Research and Reference Reagent Program. Gp120_SF2_ was also obtained as a gift from Chiron Corporation. Gp120_MN_ and the reduced and carboxymethylated (RCM) gp120_MN_ [59] were gifts from Vaxgene and Gp120_LAV-1_ was a gift from Protein Sciences, Inc.

### HIV-1 Isolates

HIV-1 syncytia inducing isolates: 92UG029, Clade A; 301596, Clade B; UG/93/086, Clade D; 92TH251, Clade E; donated by the WHO Network for HIV Isolation and Characterization were obtained from the NIH AIDS Research and Reference Reagent Program. These isolates were subsequently adapted to growth in CEM-4 cells. The cell free viral culture supernatants were stored as aliquots at −138°. Infectivity titers of these HIV-1 culture viral stocks were determined in CEM-4 cells using software (ID_50_ program version 5) developed by Dr. J. Spouge.

### Other Materials

Human lymphoblastoid cell line, CEM-T4, human HIV immune globulin (HIVIG), human MAbs IgG_1_b12 [67] and F105 [12] as well as rabbit anti-sera to human CD4 were obtained from the NIH AIDS Research and Reference Reagent Program. Recombinant soluble human CD4 was purchased from Progenics Pharmaceuticals, Inc. (Tarrytown, NY). Murine isotypic controls: IgG_1_ *κ*: anti-TNP-keyhole limpet hemocyanin and MOPC 31-C were purchased from BD Biosciences–Pharmingen (San Diego, CA) and from Sigma, respectively. (Fab)_2_ fragments of affinity purified conjugated antibodies: peroxidase-goat anti-murine IgG; peroxidase-goat anti-human IgG (H+L), R-Phycoerythrine (PE)-goat-anti-mouse IgG (H+L), Fluorescein (FITC)-goat anti-human IgG (H+L) and peroxidase conjugated goat anti-rabbit IgG were purchased from Jackson ImmunoResearch Laboratories, Inc. (West Grove, PA). PE-conjugated murine MAb to human CD4 was purchased from BD Biosciences (San Jose, CA).

### Immunization of Animals

C3H/HeJ, SWR, or C57BL/10 inbred mice were obtained from Jackson Laboratories, and BALB/c mice were obtained from Taconic. The animals were maintained according to the protocol approved by the Institutional Animal Care and Use Committee of New York University School of Medicine. Mice were immunized (4 mice per experimental group) as described previously [53] with antigens emulsified in CFA (50 µg of peptides or 10 µg of gp120_MN_) and boosted twice at three week intervals with antigens emulsified in IFA (25 µg of peptides or 10 µg of gp120_ MN_). Sera were collected prior to immunization, 2 weeks after boosts and Ab titers were determined on sera from individual mice and on pooled sera from each experimental group.

### Binding Assays

Determination of Abs binding to antigens and determination of Ab titers was performed by enzyme linked immunoadsorbent assay (ELISA) as previously described [53]. Briefly, ELISA plates were coated with most antigens at 1 µg/ml, blocked with casein in PBS, incubated with antibodies, and bound Abs were detected with the appropriate peroxidase-conjugated second antibodies. Plates were washed, substrate was added [1 mM 2,2′-azino-bis 3-ethylbenzthiazoline-6-sulfonic acid (Sigma) and 0.01% hydrogen peroxide] and after 1 h incubation the absorbance was determined at 405 nm. Antisera dilution at which an absorbance reading of 0.5 was obtained was determined by linear interpolation and the reciprocal of this dilution was taken as a titer. Binding of Abs to gp120 was assayed by capture ELISA in which gp120 was immobilized in ELISA wells using sheep antibody D7324 (Cliniqa, Inc., Fallbrook, CA) to a conserved amino acid sequence from C-terminal of gp120, that can react with both native and denatured recombinant gp120 in ELISA [58]. This assay was carried out as described elsewhere [20] with the following modifications: ELISA wells were coated with D7234 antibody dissolved in PBS, wells were blocked with 2% casein in PBS and gp120 was added to the wells in 0.2% casein, PBS. Antibodies bound to gp120 were detected as described in measurement of antibody titers.

### Competition for Antibody Binding to gp120

Ability of MCPs to inhibit binding of Abs to gp120 was tested as previously described [53]. Briefly, sera samples were incubated for 1 h at 37° with MCPs at the indicated concentrations (or without MCP–control) and Ab binding to gp120_MN_ was subsequently tested by ELISA. The appropriate dilution of sera was chosen to give an absorbance reading between 1 and 2 as determined from previous titration of Abs binding to gp120_MN_. Reduction of Ab binding was determined by comparison of binding in the presence or absence of MCP and is expressed as percent binding inhibition. Competition between Abs for binding to gp120 was determined by ELISA with gp120_MN_ captured on the plate wells. The competing Abs were added to the wells in 100 µl and plates were incubated for 1 h at 37°. Plates were subsequently washed three times to eliminate nonspecific background effects observed with preimmune sera. The biotinylated test antibody MAb 11A8 was added and its binding was determined with peroxidase conjugated streptavidin. The appropriate dilution of the test Ab was determined from a previous Ab titration of its binding to gp120 and was chosen to give absorbance reading between 1 and 2. The experimental samples were done in duplicate and the test antibody control (without the competing Abs) was done in quadruplicates. Reduction of absorbance readings in the presence of competing Abs was determined and the values for each sample were expressed as percent inhibition of Ab binding to gp120.

### Determination of Reciprocal Inhibition of Antibodies and CD4 for Binding to gp120

CD4 inhibition of Ab binding: CD4 at the appropriate concentrations was added to the ELISA wells coated with gp120_MN_ captured with Ab D7324. After 1 h incubation at room temperature antibodies were added (diluted to give an absorbance reading between 1 and 2). Subsequently plates were incubated for 1 h at 37° and Ab binding to gp120 was determined. The reduction of absorbance reading in the presence and absence of CD4 was determined and was expressed as percent inhibition of Ab binding.

Ab inhibition of CD4 binding: Abs at the appropriate concentrations were incubated for 30 minutes at 37° with gp120_MN_ captured with Ab D7324 in ELISA wells. Subsequently CD4 was added and plates were incubated for 1 hour at 37°. Both MAb 11A8 and CD4 were added in 50 µl aliquots. Bound CD4 was detected using rabbit anti-sera to human CD4 and peroxidase conjugated goat anti-rabbit IgG. CD4 concentration used determined from previous titration of CD4 binding to gp120 and was calculated to give the final absorbance value of 1–2. The reduction of absorbance reading in presence or absence of Abs was determined and expressed as percent inhibition of CD4 binding.

### Generation of Murine Hybridomas, Isolation and Characterization of Monoclonal Antibodies

C57BL/10 mice were immunized with 419–439 MCP or with the 426–441 MCP as described above. Cells from spleens of immunized mice were fused with SP2/0 meyoloma cells as described previously [68]. Hybridomas were cultured in RPMI 1640 supplemented with 20% fetal bovine serum,1% glutamine, 1% penicillin, streptomycin and 55 µM mercaptoethanol. All media components (obtained from Sigma) were hybridoma grade with the exception of mercaptoethanol (GIBCOBRL). Hybridoma supernatants were screened by ELISA using peptide immunogens or gp120_MN_. Selected hybridomas were subcloned at least 3 times. MAb 11A8 (referred to as 426 MAb) was obtained from 426–441 MCP immunized mice, while MAbs 2D3 (referred to as 419 MAb) and 4C2 were obtained from 419–439 MCP immunized mice. Monoclonal antibodies were produced in CeLLine Bioreactor flasks (Wilson Wolf Manufacturing, New Brighton, MN) by hybridomas cultured at high density in serum free medium (Sigma), purified by affinity chromatography on Sepharose coupled to protein G (Pharmacia) and dialyzed against PBS. Protein concentration in the MAb preparations was quantified using Coumassie Plus Protein Reagent, murine IgG concentration was determined using an Antibody Assay kit (both obtained from Pierce Biotechnology, Rockford, IL). Concentrations of protein and of IgG in these MAb preparations were determined to be comparable. Isotype of MAbs 11A8, 2D3 was determined to be IgG_1_ *κ* using an Isostrip from Roche Diagnostic Co. (Indianapolis, IND). MAbs were biotinylated with N-hydroxysuccinimide biotin ester (diluted in DMSO) in 0.1 M NAHCO_3_ (according to the protocol provided by Pierce Biotechnology). Biotinylated MAbs were dialyzed against PBS. The relative affinity of MAbs for rgp120, expressed as a concentration of MAb at which 50% maximal binding to gp120 was observed, was determined in ELISA with gp120 captured by D7324 antibody.

### Binding of MAbs to HIV Infected Cells

CEM-4 cells were infected with CEM-4 adapted HIV-1 in the presence of polybrene (as described above). The course of infection was followed by measurement of cell viability by trypan blue staining and loss of surface expression of CD4 (determined using PE-conjugated MAb to human CD4). To reduce nonspecific antibody binding to CEM-4 cells, cells were incubated in RPMI with 10% human serum for 1 h at 37° and washed at 4° with PBS supplemented with 2% FBS, 0.05% azide (FA buffer). Cells (2×10^5^ in 100 µl FA buffer) were incubated with Abs at the indicated concentrations. The bound Abs were detected using (as required): PE-conjugated (Fab′)_2_ fragments of goat anti-mouse IgG (H+L) or Fluorescein (FITC)-conjugated (Fab′)_2_ fragments of goat anti-human IgG (H+L). Both incubations were carried out at 4° for 30 minutes. Cells were washed after each incubation with FA buffer, fixed with 1% formaldehyde in PBS and analyzed by flow cytometry using FACScan (Becton Dickenson). Gates were set on the live cell population and fluorescence negative peak was defined on unstained cells.

### Neutralization Assay

Neutralization of SF162 pseudovirions in a single-cycle infectivity assay was measured as described [56] except that TZM-bl cells were used for virion culture. Briefly, pseudovirion suspensions were incubated for 1.5 h at 37° with Abs. These mixtures were added to the wells of 96 well plates in which TZM-bl cells were seeded in growth medium containing 1 µg/ml polybrene. After 3 day incubation cells were lysed and luciferase activity was determined in each well with Luciferase Substrate (Promega) using Lumimark Plus microplate reader. The reduction of activity was determined by comparison of the relative units of luminescence in the presence and absence of MAbs and is expressed as percent neutralization. A human anti-V3 MAb 447-52D known to have neutralizing activity [8] and a murine isotypic control anti-TNP were also included in the assays as controls.

Data presented for all experiments are representative examples of at least three replicate experiments.
